# Pilot Study Employing a Machine Learning Approach as a Potential Method for Predicting Parkinson’s Disease Using Voice as a Digital Biomarker and the SHAP Approach for Feature Engineering

**DOI:** 10.3390/bioengineering13080917

**Published:** 2026-08-13

**Authors:** Mehdi Rashidi, Syed Adil Hussain Shah, Chiara Coppola, Andrea Buccoliero, Serena Arima, Angela Lupo, Filomena My, Marta Lorenzo, Marcello Donzella, Michele Maffia

**Affiliations:** 1 Department of Mathematics and Physics “E. De Giorgi”, University of Salento, Via Lecce-Arnesano, 73100 Lecce, Italy; mehdi.rashidi@unisalento.it; 2 PolitoBioMed Lab, Department of Mechanical and Aerospace Engineering, Politecnico di Torino, 10129 Torino, Italy; syedadilhussain.shah@gpi.it; 3 Department of Research and Development (R&D), GPI SpA, 38123 Trento, Italy; andrea.buccoliero@gpi.it (A.B.); marcello.donzella@gpi.it (M.D.); 4 Department of Experimental Medicine, University of Salento, Via Lecce-Monteroni, 73100 Lecce, Italy; chiara.coppola@unisalento.it; 5 Human Science Department, University of Verona, Lungadige Porta Vittoria, 17, 37129 Verona, Italy; 6 Department of Human and Social Sciences, University of Salento, 73100 Lecce, Italy; serena.arima@unisalento.it; 7 Division of Neurology, Vito Fazzi Hospital, 73100 Lecce, Italy; angela87lupo@gmail.com (A.L.); filomenamy28@gmail.com (F.M.); martatnfp@gmail.com (M.L.)

**Keywords:** Parkinson’s disease, SHAP, explainable-AI, voice impairment, machine learning

## Abstract

**Introduction**: Voice-based digital biomarkers have emerged as a promising approach for distinguishing individuals with neurodegenerative disorders, particularly Parkinson’s disease (PD), from healthy subjects (HS). With the increasing availability of smartphone and web-based recording tools, voice data can be collected efficiently in both clinical and remote settings. However, further validation is required before such approaches can be translated into routine clinical practice. **Methods**: This study used a cross-sectional analysis at the recording level, treating repeated recordings from the same participant as separate observations collected at Vito Fazzi Hospital in Lecce, Italy. Speech recordings from individuals with Parkinson’s disease (PD) and healthy controls were collected using the dedicated Talia smartphone and web application. Sustained vowel phonation (/a/) was analyzed as the primary speech task. Following data acquisition, feature extraction was performed as a crucial step in the speech analysis pipeline, as the quality and relevance of the extracted features directly influence the ability of machine learning models to discriminate between Parkinson’s disease (PD) patients and healthy controls. To capture various aspects of speech impairment associated with PD, a comprehensive set of acoustic features was extracted, including long-term features (pitch, jitter, and shimmer), nonlinear descriptors such as Recurrence Period Density Entropy (RPDE), and short-term feature based on Mel-Frequency Cepstral Coefficients (MFCCs). These features were subsequently used to develop and evaluate machine learning models for the classification of Parkinson’s disease and healthy subjects. Feature selection was performed using SHAP to identify the most informative vocal biomarkers. Model performance was assessed using five independent random train–test splits (70% training and 30% testing), supported by an internal five-fold cross-validation procedure within the training data. Multiple machine learning models were developed and evaluated, including Random Forest, Logistic Regression, Support Vector Machine, Naive Bayes, K-Nearest Neighbors, Decision Tree, Artificial Neural Network, and Gradient Boosting. **Results**: The evaluated models demonstrated strong recording-level classification performance. Artificial Neural Networks (ANN) and K-Nearest Neighbors (KNN) achieved the highest accuracy scores (0.9545 and 0.9494, respectively), along with superior recall (up to 0.9500), precision (up to 0.9551), and F1-score (up to 0.9525). Both models also exhibited excellent discriminative ability, with ROC-AUC values reaching 0.9882 (ANN) and 0.9893 (KNN). In contrast, Naive Bayes and Decision Tree showed comparatively lower performance across all metrics. Log-loss analysis further confirmed the robustness of ANN and KNN, which achieved the lowest values (0.2552 and 0.2510, respectively), indicating well-calibrated predictions. Overall, the findings highlight the consistency and generalizability of ANN and KNN across cross-validation splits. **Conclusions**: This study demonstrates that machine learning models, particularly ANN and KNN, can effectively differentiate Parkinson’s disease from healthy conditions using voice recordings. The integration of explainable AI for feature selection enhances model transparency and clinical relevance. However, the reported performance estimates were obtained from a recording-level analysis and should be interpreted as preliminary findings. Further studies involving larger cohorts and participant-level validation strategies are required to determine the generalizability and clinical applicability of these approaches.

## 1. Introduction

Parkinson’s disease (PD) is recognized as the second most common neurodegenerative disorder after Alzheimer’s disease [1]. Although it is primarily classified as a movement disorder, PD is also characterized by a wide range of non-motor symptoms. Extensive research has been conducted in recent years to better understand its underlying molecular mechanisms and neurophysiological alterations [2]. From a pathological perspective, PD is considered a multifactorial disorder, with two key features being the intracellular accumulation of α-synuclein and the progressive loss of more than 50% of dopaminergic neurons in the substantia nigra pars compacta (SNc). While neither of these features alone is sufficient for diagnosis, both play a critical role in explaining disease mechanisms and progression. Beyond these hallmark pathological features, increasing evidence suggests that neuroinflammation, oxidative stress, mitochondrial dysfunction, and metabolic abnormalities contribute to disease progression. Furthermore, altered glucose metabolism, glycemic variability, and hypoglycemic episodes have been associated with increased neuronal vulnerability and adverse clinical outcomes in parkinsonian syndromes. These interconnected mechanisms highlight the need for reliable biomarkers capable of detecting disease-related changes at an early stage [3,4,5]. Epidemiological data indicate a substantial increase in PD prevalence, rising from approximately 2.6 million cases in 1996 to 11.8 million in 2021 [6], with projections estimating nearly 17 million cases by 2040 [1,7]. This growing burden is expected to have significant economic implications worldwide [8]. Neurodegeneration in Parkinson’s disease (PD) extends beyond dopaminergic neurons and also affects multiple neural systems, including autonomic ganglia, the spinal cord, hindbrain, basal forebrain, limbic regions, and the neocortex [9]. The motor manifestations of PD arise primarily from degeneration of the nigrostriatal pathway, leading to reduced dopamine levels in the caudate putamen. This disruption results in characteristic motor impairments such as bradykinesia and akinesia, which reflect difficulties in initiating and executing movements [10]. Rigidity, defined as increased resistance to passive movement, is another common clinical feature. Tremor presentation varies depending on the condition in which it occurs at rest, during posture maintenance, or with voluntary movement, although resting tremor is the most prevalent type. It typically occurs at a frequency of 4–6 Hz and affects approximately 70% of patients over the course of the disease [9]. Motor symptoms may present as tremor-dominant, akinesia-dominant, or mixed subtypes, often evolving and overlapping over time. In addition to motor dysfunction, PD is associated with a range of non-motor symptoms that significantly impact quality of life. Among these, anxiety disorders are observed in approximately 40–50% of patients and are linked to dysfunction of the noradrenergic system [11]. Among the socially impactful non-motor symptoms of Parkinson’s disease (PD), depression is particularly prominent and is largely associated with alterations in serotonergic and dopaminergic systems, significantly impairing patients’ quality of life [12]. Cognitive dysfunction is also common, ranging from mild cognitive decline to dementia, with reported prevalence between 20% and 80%, and includes deficits in executive function, memory, and visuospatial processing [13], Sleep disturbances affect approximately two-thirds of patients and tend to worsen as the disease progresses due to complex interactions between neurodegeneration and dopaminergic treatments [14]. dysfunction, particularly constipation, is another early non-motor manifestation, affecting nearly 63% of patients and often appearing years before motor symptoms. This is primarily attributed to pathology within the enteric nervous system and disrupted gut-brain interactions [15,16]. Speech impairment is one of the most prevalent manifestations of Parkinson’s disease (PD), arising from disrupted neural control of speech production and resulting in abnormalities in voice quality, articulation, prosody, and speech timing [17,18]. These alterations can occur during the early stages of the disease and therefore represent promising digital biomarkers for PD detection. Common speech-related abnormalities include reduced vocal intensity and pitch variability (hypophonia), increased jitter and shimmer, altered spectral characteristics, and irregular speech pacing [19]. Furthermore, hypokinetic dysarthria affects approximately 90% of patients and may precede overt motor symptoms, highlighting the potential of voice analysis as a non-invasive and objective tool for early Parkinson’s disease identification [20]. A wide range of acoustic and nonlinear speech features has been investigated for PD detection, including fundamental frequency (F0), formant frequencies, Mel-frequency cepstral coefficients (MFCCs), jitter, shimmer, and recurrence period density entropy (RPDE). These features capture complementary aspects of speech production, vocal fold dynamics, and signal irregularity, making them valuable candidates for machine learning-based classification. In particular, MFCCs effectively characterize the spectral properties of speech signals and provide robust representations that are less sensitive to background noise, while RPDE offers additional insights into vocal fold dynamics and nonlinear signal behavior associated with PD [21]. Although long-term features tend to be more stable, their high intercorrelation may negatively affect classification performance. Therefore, combining long-term and short-term features is often advantageous. In addition, non-linear measures such as recurrence period density entropy (RPDE) offer deeper insights into vocal fold dynamics and signal irregularities associated with PD [22]. Machine learning (ML), a core domain of artificial intelligence, enables the analysis of complex, high-dimensional data for disease classification [23]. In the context of PD, ML models trained on labeled datasets using supervised learning techniques have demonstrated strong potential for identifying patients based on voice characteristics [24]. A variety of algorithms including k-nearest neighbors, decision trees, support vector machines, Naïve Bayes, logistic regression, gradient boosting, artificial neural networks, and random forests have achieved high predictive performance by capturing intricate relationships between acoustic features and disease status. However, despite their effectiveness, many ML models lack interpretability, which limits their adoption in clinical practice. Explainable artificial intelligence (XAI) addresses this limitation by enhancing model transparency and interpretability in medical applications [25]. Among XAI techniques, SHapley Additive exPlanations (SHAP) has emerged as a widely used, model-agnostic, game-theoretic approach for quantifying feature importance [26]. SHAP enables the evaluation and visualization of individual feature contributions to model predictions, facilitating the identification of the most informative acoustic biomarkers for distinguishing PD from healthy conditions. Such interpretability is essential in clinical settings to ensure the reliability and validity of model outputs [27]. In this study, we focus on the application of machine learning approaches for the classification of Parkinson’s disease, five independent random train–test splits (70% training and 30% testing) combined with internal five-fold cross-validation within the training data. By integrating SHAP methods to select strongly relevant features associated with Parkinson’s voices, we improve model interpretability and achieve high classification performance. According to metrics such as precision, recall, and ROC-AUC, we could achieve the best model for automatic detection of Parkinson’s disease from voice using speech impairment.

## 2. Materials and Methods

**Figure 1:**
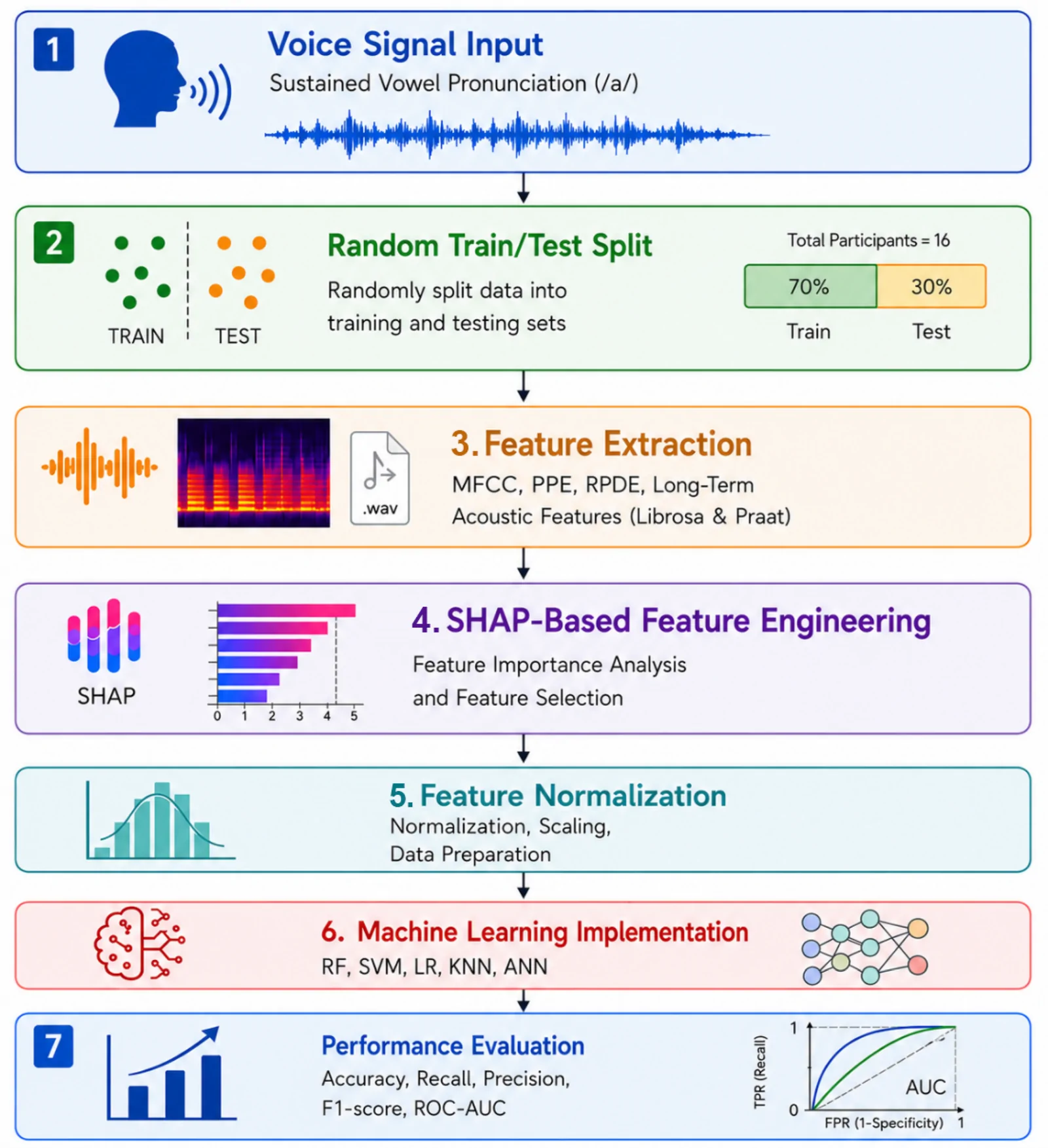
Machine learning pipeline for Parkinson’s disease detection using sustained vowel (/a/) pronunciation, Random train–test splits, SHAP-based feature engineering, and performance evaluation.

In this study, a combination of hardware and software tools was employed to facilitate the acquisition and analysis of voice recordings from individuals with Parkinson’s disease (PD) and healthy controls (HC). During the initial data collection phase, voice recordings were obtained in a controlled environment at Vito Fazzi Hospital in Lecce, Italy, using an IsoVox vocal booth and a RØDE microphone. Participants were instructed to produce the sustained vowel phonation (/a/), which served as the primary speech task for acoustic analysis. The present study employed recording-level validation rather than participant-level validation. Repeated recordings from the same participant were treated as independent observations, and participant-level cross-validation was not performed. In addition, the study was designed as a cross-sectional analysis at the recording level and consisted of seven main stages, as illustrated in Figure 1. First, the recorded voice signals were collected and organized for analysis. Second, the voice recordings were partitioned into five independent random train–test splits, with 70% of the recordings allocated to the training set and 30% to the testing set. Third, acoustic feature extraction was performed using Librosa, Praat, and Parselmouth to derive long-term acoustic features, Mel-Frequency Cepstral Coefficients (MFCCs), and Recurrence Period Density Entropy (RPDE). Fourth, SHAP-based feature engineering was applied to identify and select the most informative acoustic biomarkers. Fifth, feature normalization and data preprocessing procedures were conducted to prepare the datasets for machine learning analysis. Sixth, the selected features were used to develop and evaluate multiple machine learning models, including Random Forest (RF), Support Vector Machine (SVM), Logistic Regression (LR), K-Nearest Neighbors (KNN), Artificial Neural Networks (ANN), Decision Trees (DT), Naïve Bayes (NB), and Gradient Boosting (GB). Finally, model performance was assessed using accuracy, precision, recall, F1-score, and ROC-AUC metrics.

### 2.1. RØDE Microphone Description

The RØDE NT-USB is a high-quality USB condenser microphone manufactured in Sydney, Australia, designed for direct digital audio recording without requiring an external audio interface. It incorporates a large-diaphragm condenser capsule along with a built-in analog-to-digital converter (ADC) and USB connectivity, allowing seamless integration with computers and mobile devices for high-fidelity sound capture [28].

### 2.2. Data Storage and Management

After acquisition, all voice recordings were securely transferred to POHEMA website [29], the centralized server platform developed and managed by GPI S.p.A company (Trento, Italy). This infrastructure enabled systematic data storage, remote monitoring, and secure handling of clinical voice recordings throughout the study period. All voice recordings were downloaded from the server. Each participant had a unique identifier, referred to as a tax code (Codice Fiscale in Italian), which enabled the identification and tracking of individual recordings.

### 2.3. Data Collection

The study was conducted in accordance with the Declaration of Helsinki and other relevant guidelines and regulations. Informed consent was obtained from all participants and/or their legal guardians. The protocol was reviewed and approved by the Lecce Ethics Committee (Institutional Ethics Committee of the Vito Fazzi Hospital, Lecce, Italy) with the approval number 17032/2023 (Identficazione di feature predittivi/prognostici mediante l’analisi del parlato in soggetti affetti da Parkinson).

The dataset comprised recordings from 8 patients diagnosed with Parkinson’s disease (PD) and 8 healthy subjects (HS), as shown in Table 1. Each participant produced a sustained vowel (/a/) with durations ranging from 6 to 16 s. Audio signals were recorded at a sampling rate of 22,050 Hz and stored as 32-bit floating-point WAV files. Data collection followed a cross-sectional analysis at the recording level over a six-month period to capture voice changes associated with disease progression. In total, 96 recordings were obtained from healthy subjects and 93 from PD patients; 1 PD participant contributed only 9 recordings instead of 12 due to completing 4.5 months of follow-up [30]. For PD patients, the initial recording session was conducted at Vito Fazzi Hospital using the TALIA-WEB platform developed by GPI company, a high-quality RØDE microphone, and an IsoVox vocal booth (Halmstad, Sweden) to minimize environmental noise (The Stand Set configuration includes a height-adjustable speaker stand (model: Millenium BS2011 MK II; Thomann GmbH, Burgebrach, Germany)). Participants were recruited through the POHEMA platform (GPI server). Subsequent recordings were performed remotely at home using the TALIA-APP mobile application, with two sessions per month. Healthy subjects followed the same mobile-based protocol, with the exception that only PD patients underwent their first recording session in the hospital setting. Patient selection for the PD group was performed by a neurologist at Vito Fazzi Hospital based on standard clinical criteria, including the Unified Parkinson’s Disease Rating Scale (UPDRS) and Hoehn and Yahr staging [31].

### 2.4. Voice Preprocessing

The collected WAV audio files were first processed using Audacity (version 3.7.0) to reduce background noise. Filtering was applied using predefined floor and ceiling thresholds, set at 75 dB and 300 dB for male participants and 100 dB and 600 dB for female participants. Subsequently, all signals were normalized within the range of [−1, 1]. Initial and trailing silent portions of each recording were removed using a short-time energy threshold within a sliding window. The processed audio files were then further refined in Praat (version 6.4.34) to eliminate any residual silence and ensure readiness for subsequent analysis. Following preprocessing, 96 recordings from healthy subjects and 93 from Parkinson’s disease (PD) patients were selected for segmentation. Due to variations in recording duration, each audio file was divided into uniform segments of 2.5 s using Python, yielding a total of 777 segments. This segmentation strategy effectively increased the dataset size and contributed to reducing variability during model training and analysis [30].

### 2.5. Random Train–Test Split

**Figure 2:**
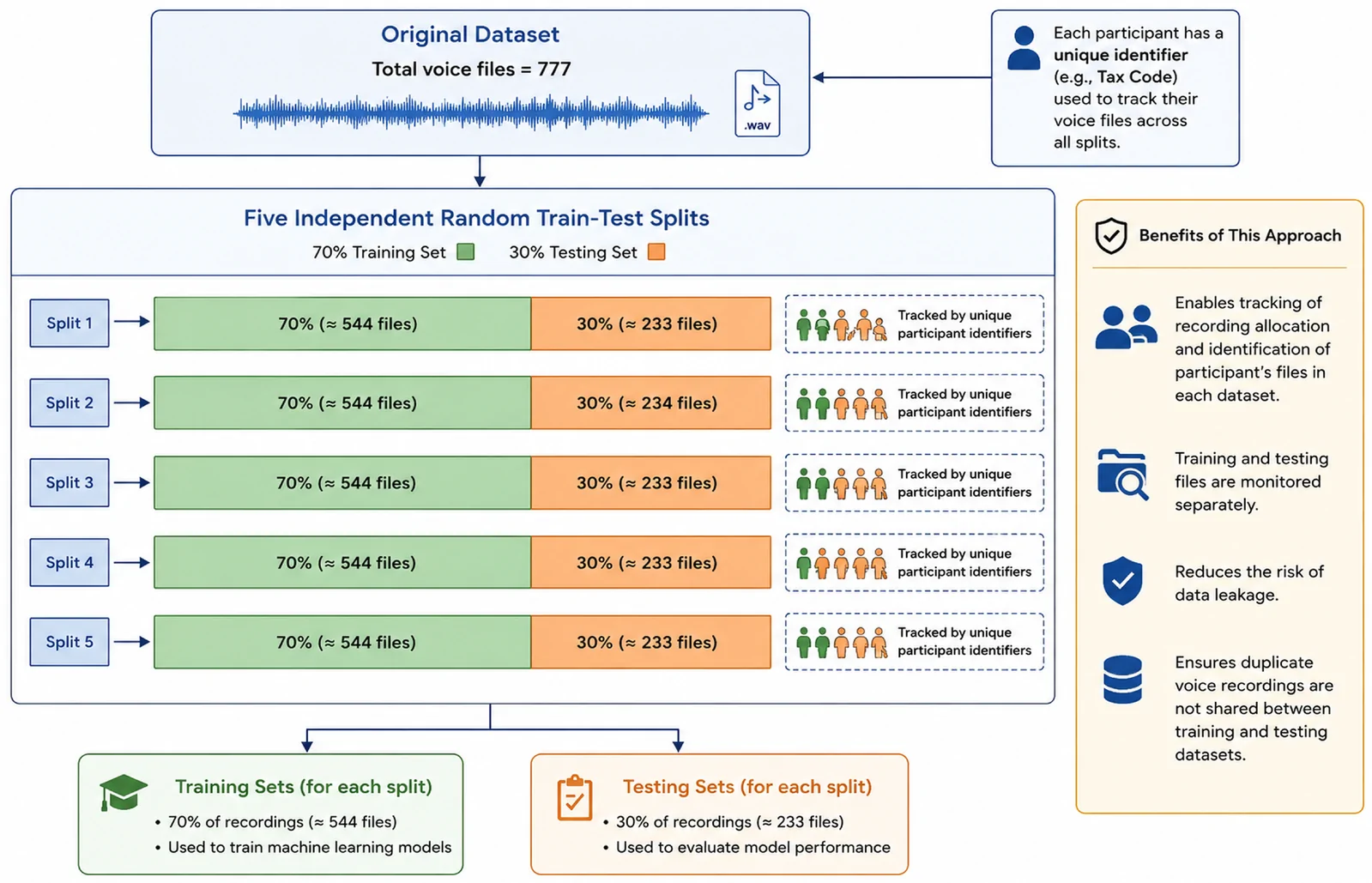
Random train–test Splits (70% train vs. 30% test).

Before feature extraction, the voice recordings were divided into training and testing datasets using five independent random train–test splits, with 70% of the recordings allocated to the training set and 30% to the testing set. After the train–test split, segmentation was performed using non-overlapping 2.5-s windows, resulting in a total of 777 voice segments. Because the original recording durations varied across participants, the number of segments per participant was calculated and reported in Table 2. As shown in Table 2, the number of segments per participant ranged from 30 to 67 segments. To evaluate class-level balance, the distribution of HS and PD segments was also calculated for the training and testing datasets across the five random splits. As shown in Table 3, all five splits maintained the same class-level distribution, with 544 training segments and 233 testing segments, corresponding to an approximate 70:30 train–test ratio. The training set included 283 HC and 261 PD segments, while the test set included 121 HC and 112 PD segments. This splitting strategy enabled the allocation of recordings to be tracked using unique participant identifiers, allowing the training and testing files to be monitored separately and reducing the risk of file-level data leakage, as illustrated in Figure 2.

### 2.6. Sensitivity Analysis for Demographic Confounding

To evaluate the potential confounding effect of age and sex, a participant-level sensitivity analysis was performed using logistic regression with age and sex as the only predictors. Since age and sex are participant-level variables, each participant was included only once, resulting in 16 observations. The model was evaluated using leave-one-participant-out validation.

### 2.7. Feature Extraction and Feature Engineering

Although acoustic features can be extracted using software packages such as Praat (version 6.4.34) and the Parselmouth library in Python, this study primarily used Parselmouth to extract long-term acoustic features, including jitter, shimmer, and pitch-related measures. In addition, Mel-Frequency Cepstral Coefficients (MFCCs) and non-standard nonlinear features, such as Recurrence Period Density Entropy (RPDE), were extracted from the voice recordings. Following feature extraction, SHAP (Shapley additive explanation) was employed to evaluate feature importance and identify the most informative vocal biomarkers for machine learning classification.

#### 2.7.1. Acoustic and Nonlinear Voice Feature Description

Acoustic characteristics commonly associated with vocal fold behavior during sustained vowel production were computed. These included the mean and standard deviation of F0, along with jitter and shimmer. F0 refers to the rate at which the vocal folds open and close during voiced speech, essentially capturing the pitch of the voice. Jitter quantifies how much F0 fluctuates from one cycle to the next, while shimmer reflects how much the amplitude of the waveform varies over time. All measurements were derived from recordings of a sustained ‘a’ vowel, and a total of 20 acoustic features were extracted for use in the analysis [32]. Recurrence Period Density Entropy (RPDE) (nonlinear feature) is a measure used to assess how consistent or rhythmic a voice signal is over time. In people without vocal impairments, the vocal folds tend to vibrate in a stable, predictable manner. However, conditions like Parkinson’s disease can interfere with this regularity, causing erratic or uneven vocal fold movement that results in a less controlled voice output. RPDE works by examining how often comparable patterns repeat themselves throughout the signal. Its values are scaled on a range from 0 to 1, where scores near 0 reflect a high degree of consistency and scores approaching 1 suggest increasing levels of instability. As such, this measure serves as a useful tool for detecting vocal abnormalities and evaluating the smoothness of vocal fold vibration [33]. Linear Predictive Coding (LPC) is a speech-processing technique that models the vocal tract by representing each speech sample as a linear combination of previous samples. LPC-based analysis is widely used in speech and voice research because it provides information about vocal tract resonances and formant structure. In Parkinson’s disease, impairments in motor control may alter articulatory precision and vocal tract dynamics, making LPC-derived representations useful for characterizing pathological speech patterns [30]. Mel-Frequency Cepstral Coefficients (MFCCs) were originally developed for automatic speech recognition applications and have since become one of the most widely used acoustic features in speech and voice analysis [34]. In recent years, MFCCs have been extensively applied in the detection of voice disorders, including Parkinson’s disease. As short-term spectral features, MFCCs provide information that is complementary to conventional long-term acoustic measures, thereby enhancing the discriminatory power of classification models. MFCC values reflect the distribution of spectral energy across frequency bands on the Mel scale; more negative coefficients generally indicate a greater concentration of energy in higher-frequency regions of the spectrum. Since Parkinson’s disease is frequently associated with vocal abnormalities such as hoarseness, breathiness, and reduced phonatory control, alterations in high-frequency energy patterns may be captured by MFCC features, making them valuable biomarkers for distinguishing affected individuals from healthy controls [35]. The key parameters used for feature extraction are summarized in Table 4.

#### 2.7.2. SHAP (Shapley Additive Explanation)

SHAP (Shapley additive explanation) provides an interpretable framework for quantifying the contribution of each feature to model predictions. Specifically, SHAP values indicate both the magnitude and direction of a feature’s influence on the classification outcome. Features with larger absolute SHAP values exert a greater impact on the model’s decision-making process. Furthermore, the SHAP summary plot illustrates how high and low feature values affect the probability of classifying a recording as belonging to a Parkinson’s disease patient or a healthy control. Based on SHAP evaluation, 20 features were selected to construct the final dataset for machine learning implementation (Table 5). Table 5 summarizes the acoustic features identified as the most relevant predictors according to SHAP analysis. The selected features included short-term feature (MFCCs), long-term features (jitter, shimmer, pitch-related features), and nonlinear characteristics such as RPDE, reflecting multiple aspects of speech production affected by Parkinson’s disease.

**Figure 3:**
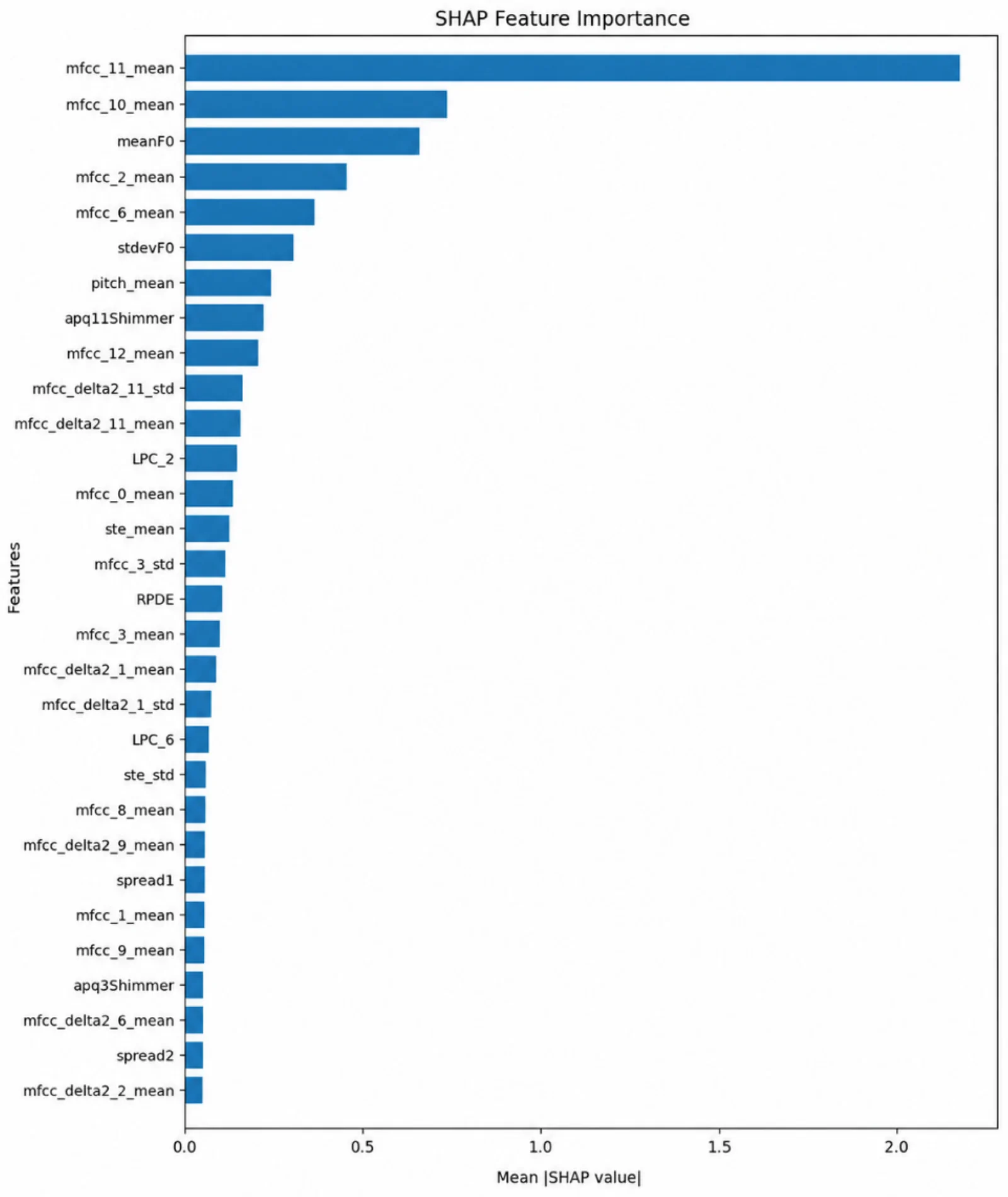
SHAP feature importance ranking of the extracted acoustic features. Features are ordered according to their mean absolute SHAP values, indicating their overall contribution to the machine learning model predictions. Higher SHAP values represent greater influence on the classification of Parkinson’s disease and healthy subjects.

Figure 3 presents the global SHAP feature importance ranking based on the mean absolute SHAP values of the extracted acoustic features. The results indicate that MFCC_11_mean exhibited the highest contribution to model predictions, followed by MFCC_10_mean and meanF0. The prominence of MFCC-related features highlights the importance of spectral information in capturing Parkinson’s disease-related speech alterations. In addition, voice-quality measures such as pitch_mean, stdevF0, apq11Shimmer, and RPDE also demonstrated meaningful contributions to the classification process. These findings suggest that both spectral and phonatory characteristics are important for distinguishing Parkinson’s disease patients from healthy controls.

**Figure 4:**
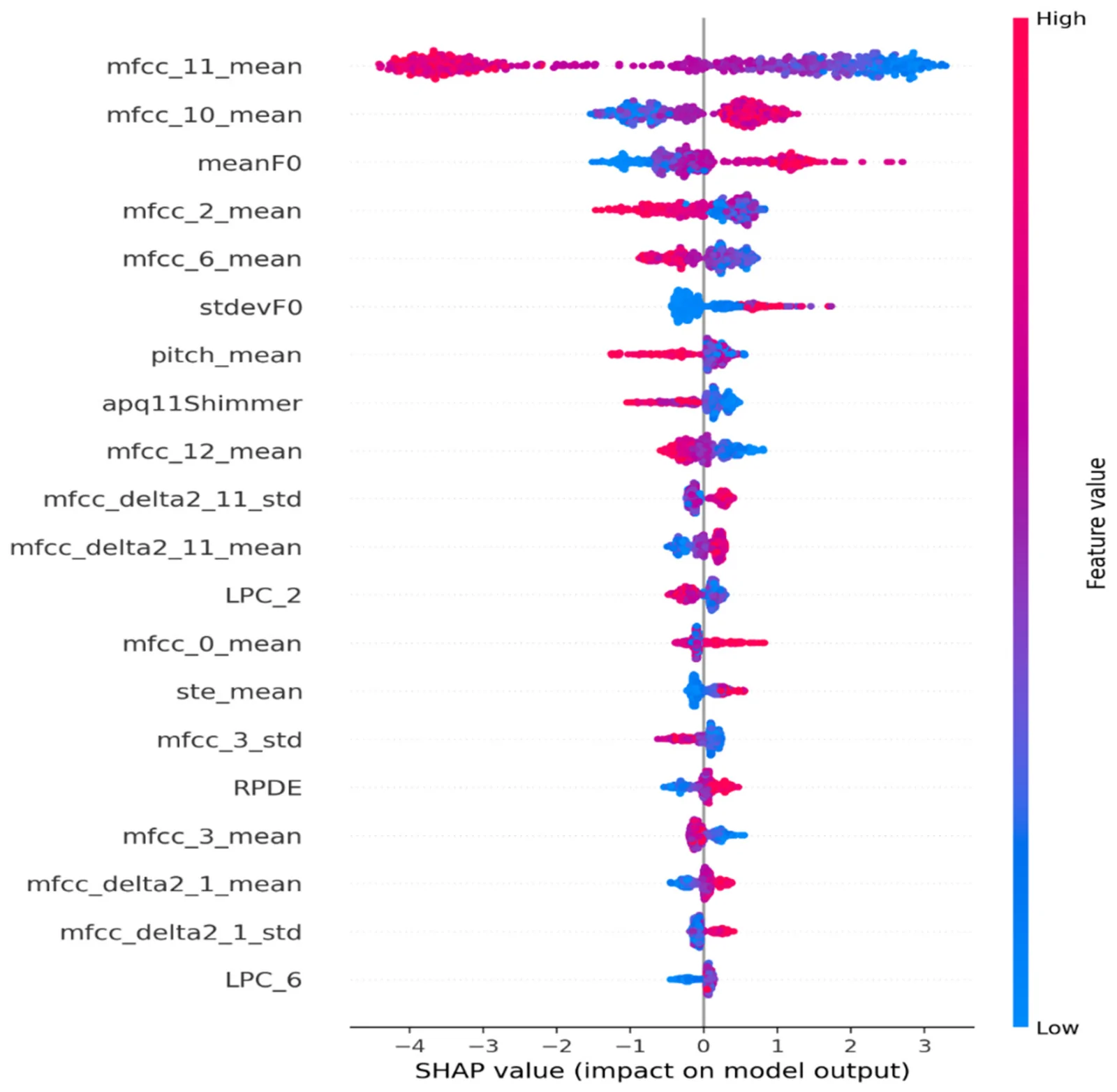
SHAP summary plot illustrating the contribution and ranking of acoustic features in Parkinson’s disease classification.

Figure 4 provides a more detailed interpretation of feature behavior through the SHAP summary plot. Unlike Figure 3, which shows only overall feature importance, the SHAP summary plot illustrates both the magnitude and direction of each feature’s contribution to individual predictions. The color distribution indicates whether high or low feature values increase or decrease the probability of classification as Parkinson’s disease. For example, several highly ranked MFCC features demonstrated consistent SHAP patterns, indicating that changes in their values strongly influenced model outputs. The wide spread of SHAP values observed for MFCC_11_mean, MFCC_10_mean, and meanF0 further confirms their substantial impact on prediction performance. Overall, the SHAP summary plot provides additional evidence that specific spectral and voice-quality features contribute differently across participants, supporting the interpretability and transparency of the proposed machine learning framework. SHAP, as an explainable artificial intelligence (XAI) method, provides interpretable insights into feature importance and supports transparent feature selection [36].

### 2.8. Preprocessing of Machine Learning (ML)

The dataset was initially divided into five independent random train–test splits, with 70% of the samples allocated to the training set and 30% to the testing set. For each split, acoustic features were extracted from the voice recordings, and SHAP-based feature selection was performed exclusively on the training data to identify the most informative vocal biomarkers. The resulting feature subset was subsequently applied to the corresponding test set. Following feature selection, the datasets were imported into Python using the Pandas library for further analysis. Preprocessing steps included data loading, conversion to matrix format, and feature normalization. An internal five-fold cross-validation procedure was conducted within each training set for model development and hyperparameter optimization. Each train–test split was processed independently, and the final performance metrics were reported as the average across the five independent splits [37].

### 2.9. Hyper-Parameter Tuning

To optimize model performance and ensure fair comparison across classifiers, hyperparameter tuning was performed for each machine learning algorithm. The final hyperparameter configurations were selected based on cross-validation performance within the training folds. As an example, the Random Forest classifier was implemented with 400 decision trees, no restriction on maximum tree depth, a minimum of 5 samples required to split an internal node, and a minimum of 1 sample per leaf. Other hyperparameters are presented in Table 6.

### 2.10. Classification Algorithms of ML

At this stage, the dataset was ready for implementation with various machine learning models, including Random Forest (RF), Logistic Regression (LR), Support Vector Machine (SVM), Naive Bayes (NB), k-Nearest Neighbors (KNN), Decision Tree (DT), Artificial Neural Network (ANN), and Gradient Boosting (GB). For each model, hyperparameter optimization was performed to identify the best-performing configurations, which are reported in Table 6. Hyper-parameter tuning plays a critical role in optimizing model performance and is therefore an essential step when implementing machine learning algorithms.

### 2.11. Cross-Validation

In addition to the five independent random train–test splits, an internal five-fold cross-validation procedure was performed within each training set to reduce the risk of overfitting and improve model generalization. For each train–test split, the training data were further partitioned into five folds. During each iteration, four folds were used for model training and one fold was used for validation. This process was repeated five times, allowing each fold to serve once as the validation set. The average validation performance across the five folds was used to guide model development and hyperparameter optimization. After completion of the internal cross-validation procedure, the final model was trained on the entire training set and subsequently evaluated on the independent test set [37].

### 2.12. Evaluation Criteria

Model performance was evaluated using accuracy, precision, recall, F1-score, and the area under the receiver operating characteristic curve (ROC–AUC). These metrics were calculated according to their standard mathematical definitions presented in Section 1. Accuracy measures the overall proportion of correctly classified samples, precision quantifies the reliability of positive predictions, recall (sensitivity) assesses the ability of the model to correctly identify Parkinson’s disease cases, and the F1-score provides a balanced measure of precision and recall. In addition, ROC–AUC was used to evaluate the overall discriminative ability of each model across different classification thresholds. These metrics were used to compare the performance of the machine learning models and identify the most effective approach for distinguishing Parkinson’s disease patients from healthy controls [38].

## 3. Results

The experiments were conducted using Jupyter Notebook (version 7.0.8) and Google Colab environments on a Windows system, utilizing the hardware specifications listed in Table 7:

This six-month voice recording study initially included 11 patients with Parkinson’s disease (PD) and 11 healthy subjects (HS), recruited at Vito Fazzi hospital in Lecce (Italy), with approval from the Lecce Ethics Committee (registration number: 17032/2023). Due to participant attrition, the final analysis was conducted on a reduced dataset comprising 8 PD patients and 8 healthy subjects, yielding a total of 93 and 96 voice recordings, respectively.

Based on Table 8, the demographic and clinical characteristics of the participants were analyzed in this study. Both male and female individuals were included in the recruitment process. In the Parkinson’s disease (PD) group, the gender distribution was balanced (50% male and 50% female), whereas in the healthy control (HC) group, males outnumbered females by a ratio of approximately three to one. All patients in the PD group exhibited voice impairments, which aligns with the known impact of Parkinson’s disease on speech production. Furthermore, the mean age of participants in the PD group was above 72 years, indicating that the study population primarily consisted of elderly individuals, consistent with the typical age of PD onset and progression. As shown in Table 9, the age- and sex-only logistic regression model achieved an AUC of 0.844, an accuracy of 0.750, and a balanced accuracy of 0.750. These results indicate that demographic variables had substantial discriminatory power in this small cohort. Therefore, the high performance of the acoustic models should be interpreted cautiously, as age-related vocal changes may have contributed to the classification results.

This section presents the results obtained from the machine learning analysis, along with a comprehensive description of the evaluation metrics used to assess model performance. The effectiveness of each model was evaluated using multiple performance indicators, including accuracy, recall, precision, F1-score, ROC–AUC, and log-loss. These metrics provide complementary perspectives, ensuring a robust and reliable assessment of classification performance, particularly in a medical diagnostic context. A diverse set of machine learning algorithms was implemented and systematically evaluated, including Random Forest (RF), Logistic Regression (LR), Support Vector Machine (SVM), Naive Bayes (NB), K-Nearest Neighbors (KNN), Decision Tree (DT), Artificial Neural Network (ANN), and Gradient Boosting (GB). To improve model efficiency and reduce redundancy, feature selection was performed using the SHAP (SHapley Additive exPlanations) method, retaining only the most informative features. To minimize overfitting and ensure generalizability, an random train–test splits strategy with k=5 was employed. In each split, 70% of the data were used for training, while the remaining 30% were reserved for testing. In addition, an internal 5-fold cross-validation procedure was applied within the training set to further optimize model performance. The final evaluation of all machine learning models was conducted by aggregating performance metrics across the five cross-validation splits, allowing for the identification of the most robust classifier for distinguishing between Parkinson’s disease (PD) and healthy subjects (HS). Based on the averaged results, Artificial Neural Networks (ANN) and K-Nearest Neighbors (KNN) consistently demonstrated superior performance across all evaluation metrics. Specifically, ANN achieved the highest overall accuracy (0.9545), closely followed by KNN (0.9494). In contrast, Naive Bayes (NB) and Decision Tree (DT) exhibited the lowest accuracy values, suggesting limited suitability for this classification task. From a clinical perspective, recall (sensitivity) is a critical metric, as it reflects the ability of a model to correctly identify patients with Parkinson’s disease. Both ANN and KNN achieved the highest recall values, indicating strong sensitivity to PD cases. Conversely, NB and DT showed relatively lower recall values (0.7714 and 0.8321, respectively), suggesting a higher risk of misclassifying PD patients as healthy. A similar trend was observed for precision and F1-score, where ANN and KNN again achieved the highest values (up to 0.9551), reinforcing their reliability and stability. The ROC–AUC metric, which measures the model’s ability to discriminate between classes across different thresholds, further confirmed these findings. KNN achieved the highest ROC–AUC (0.9893), followed closely by ANN (0.9882), indicating excellent separability between PD and HS groups. In contrast, NB and DT recorded the lowest ROC–AUC values among the evaluated models. Analysis of log-loss provided additional insight into model calibration and prediction confidence. Lower log-loss values indicate more accurate and well-calibrated probabilistic predictions. KNN and ANN achieved the lowest log-loss values (0.2510 and 0.2552, respectively), demonstrating high confidence and reliability. On the other hand, the Decision Tree model produced the highest log-loss (2.8687), indicating poor probability calibration and less reliable probabilistic predictions.

**Figure 5:**
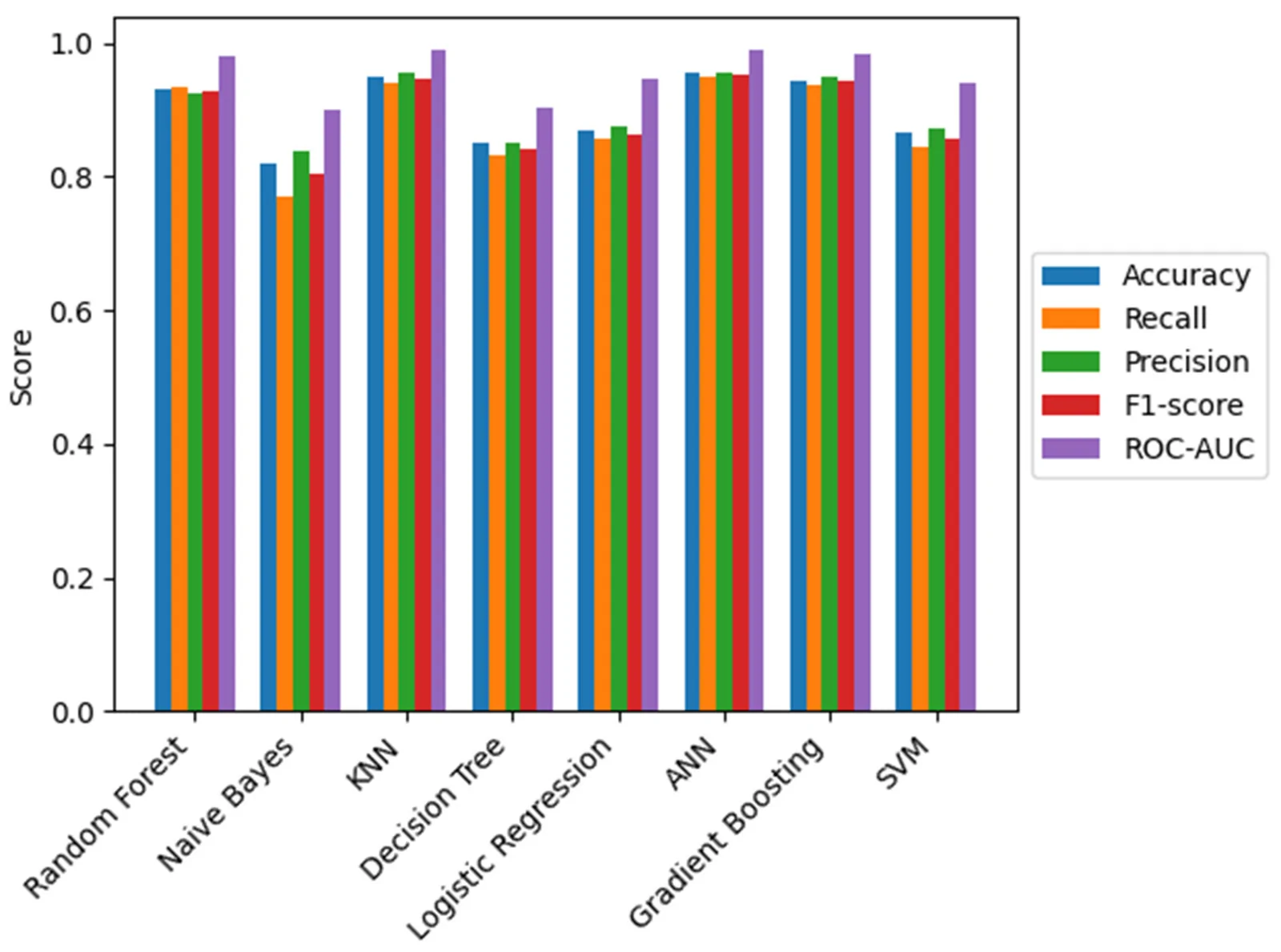
Grouped bar chart comparing the performance of machine learning models across evaluation metrics (accuracy, recall, precision, F1-score, and ROC–AUC) on the test set, averaged over five cross-validation splits.

Overall, as summarized in Table 10, ANN and KNN emerged as the most effective models for this study due to their high accuracy, strong sensitivity, excellent discriminative ability, and low error rates across all cross-validation splits. To complement the numerical results, a grouped bar chart (Figure 5) was generated to visually compare the performance of all models across the evaluated metrics. This graphical representation facilitates intuitive comparison and highlights performance differences more clearly than tabular values alone. As shown in Figure 5, ANN and KNN consistently outperform the other models across all metrics, while NB and DT remain comparatively weaker. The visualization reinforces the conclusions drawn from Table 10 and provides an accessible summary of model performance for readers.

## 4. Discussion

In this study, the potential of machine learning (ML) methods was evaluated for detecting Parkinson’s Disease (PD) vs. Healthy Subject (HS) classification based on vocal biomarkers. The goal was to propose and implement a reliable and reproducible classification system capable of automatic diagnosis of Parkinson’s disease. The results of this study reveal that the application of ML techniques provided an outstanding discriminative ability on the basis of evaluation metrics, thus highlighting the effectiveness of ML models in voice-based Parkinson’s detection. The presented results of ML models application correspond with the findings of other studies supporting the effectiveness of voice-based detection of Parkinson’s disease via machine learning techniques.

Iyer et al. [30] reported a high accuracy of Parkinson’s detection based on analysis of acoustic biomarkers of voice via ML methods. Nevertheless, their study had a cross-sectional design and included 40 patients with Parkinson’s disease and 41 healthy subjects, with each participant contributing only a limited number of voice recordings collected during a single assessment session. In contrast, although our analysis was also conducted at the recording level, voice data were collected repeatedly over a six-month period using a standardized acquisition protocol. This approach resulted in 93 voice recordings from Parkinson’s disease patients and 96 recordings from healthy subjects, originating from 8 PD patients and 8 healthy participants. Furthermore, each recording exceeded 6 s in duration, providing richer acoustic information for feature extraction and machine learning analysis. Another strength of our study was the use of the TALIA smartphone and web-based platform developed by GPI S.p.A. (Italy), which ensured standardized voice acquisition across recording sessions and reduced variability associated with data collection procedures. In addition, SHAP-based feature selection enabled the identification of highly informative acoustic biomarkers, potentially contributing to the improved classification performance observed in the present study.

One of the pioneering studies on Parkinson’s disease and vocal impairment classification was carried out by Little et al. [33]. One of the pioneering studies investigating the classification of Parkinson’s disease based on vocal impairments was conducted by Little et al. [37]. Using sustained vowel phonations and a Support Vector Machine (SVM) classifier, the authors achieved an overall classification accuracy of 0.914 by combining non-standard dysphonia measures, including Pitch Period Entropy (PPE), Recurrence Period Density Entropy (RPDE), Detrended Fluctuation Analysis (DFA), and Harmonics-to-Noise Ratio (HNR). However, their dataset consisted of 23 Parkinson’s disease patients and only 8 healthy controls, resulting in a notable class imbalance that may have influenced classification performance. In addition, their feature set did not include Mel-Frequency Cepstral Coefficients (MFCCs), which are widely recognized as short-term feature for speech analysis.

In contrast, our study combined short-term features, MFCC-based spectral descriptors, and nonlinear nonstandard features within a unified machine learning framework. Furthermore, SHAP-based feature selection enabled the identification of the most informative acoustic biomarkers while improving model interpretability. The SHAP analysis consistently identified several MFCC-derived features among the strongest predictors, highlighting the importance of spectral information for Parkinson’s disease detection. Consequently, classifiers such as KNN and ANN achieved higher performance metrics than those reported by Little et al., suggesting that the integration of explainable feature selection and a broader acoustic feature set may enhance the discrimination between Parkinson’s disease patients and healthy controls.

Shen et al. [36] recently proposed an explainable artificial intelligence framework for Parkinson’s disease detection using voice recordings and a hybrid deep learning architecture combining MLP, CNN, RNN, and MKL. Their model achieved an accuracy of 0.9111 and an AUC of 0.9125 using a dataset of 81 voice recordings obtained from 40 Parkinson’s disease patients and 41 healthy controls. Unlike their study, which relied on a single voice recording per participant from a publicly available dataset, our study collected repeated voice recordings over a six-month period, resulting in 93 recordings from Parkinson’s disease patients and 96 recordings from healthy controls. This approach enabled the collection of a substantially larger amount of voice data and better reflected real-world variability in speech production. Furthermore, while Shen et al. focused primarily on developing a hybrid deep learning framework and model explainability, our study combined short-term feature, MFCC-based descriptors, and nonlinear voice measures with SHAP-based feature selection to identify the most informative vocal biomarkers. The superior classification performance observed in our models may be partially attributed to the feature-selection strategy, the standardized recording protocol implemented through the TALIA platform, and the larger recording-level dataset available for training and evaluation. In our earlier study [1], we analyzed the dataset of Iyer et al. [30] and applied machine learning methods to analyze short-term, long-term, and non-standard features. According to the reported performance metrics, random forest and SVM provided the best results, and the worst result was produced by KNN. On the contrary, in our current study, KNN achieved quite a high score compared with the earlier experiment. It can be explained by SHAP-based feature selection that allowed us to detect highly relevant features.

Eri Kiyoshige et al. [39] demonstrated that voice biomarkers combined with artificial intelligence can effectively identify cognitive impairment in community-dwelling adults, achieving an AUC of 0.89 in a large cross-sectional cohort. Similar to our findings, their results support the growing role of speech as a non-invasive digital biomarker for neurological disorders. Both studies highlight the potential of voice-based machine learning systems for remote screening, early detection, and longitudinal monitoring through easily obtainable speech recordings. While Kiyoshige et al. focused on cognitive impairment using deep-learning-derived voice embeddings, our study specifically investigated Parkinson’s disease using interpretable acoustic, spectral, and nonlinear vocal features selected through SHAP analysis. The high performance achieved in both studies suggests that voice contains disease-specific information that can be exploited by machine learning algorithms to support neurological assessment. Collectively, these findings strengthen the evidence that voice-based artificial intelligence may become an important component of future digital health platforms for the screening and monitoring of neurodegenerative disorders.

Suppa et al. [31] investigated voice abnormalities in Parkinson’s disease using machine learning techniques in a large and clinically well-characterized cohort comprising 115 patients with Parkinson’s disease and 108 healthy controls. Their study demonstrated that voice impairment is detectable even in the early stages of Parkinson’s disease, worsens with disease progression, and is partially responsive to levodopa treatment. Furthermore, they reported significant correlations between machine-learning-derived voice measures and clinical indicators of disease severity, including UPDRS and Hoehn and Yahr scores. Our findings are consistent with these observations, further supporting the utility of voice as a digital biomarker for Parkinson’s disease. However, several methodological differences should be noted. Suppa et al. employed a support vector machine classifier and extracted acoustic features using the OpenSMILE framework, whereas our study combined acoustic biomarkers, MFCC features, nonlinear voice measures, and SHAP-based explainable feature selection to identify the most informative predictors. In addition, while their analysis was based on a large cross-sectional cohort assessed at a single clinical time point, our dataset consisted of repeated voice recordings collected over a six-month period using a standardized smartphone-based platform, generating 189 recordings (96 healthy control and 93 Parkinson’s disease recordings). This approach enabled the collection of a larger number of speech samples per participant and may better reflect variability encountered in real-world remote monitoring environments. The high classification performance achieved in both studies highlights the robustness of voice-based machine learning approaches and further supports their potential application in digital health and telemonitoring of Parkinson’s disease.

Wroge et al. [40] used 5-fold cross-validation and reported that Gradient Boosted Decision Trees provided the highest ROC-AUC and Accuracy when working with the AVEC and GeMAPS feature sets. Decision Trees provided the poorest results for AVEC, and Gradient Boosting gave the worst performance for GeMAPS. In our study, we achieved higher performance for AVEC and equal for GeMAPS because of using SHAP-based feature selection.

Hawi et al. [18] investigated Parkinson’s disease detection using a dataset divided into training, validation, and testing subsets and employed a Random Forest classifier trained on a combination of long-term acoustic features and MFCCs. Their findings demonstrated that integrating these complementary feature sets improved classification performance, achieving an accuracy of 0.8884. In agreement with their results, our study also highlights the effectiveness of combining diverse acoustic biomarkers for Parkinson’s disease detection. However, unlike Hawi et al., who relied on a single machine learning algorithm, our study systematically compared multiple classifiers, including KNN, Random Forest, SVM, ANN, and Gradient Boosting. This broader evaluation provided a more comprehensive assessment of model performance and robustness, enabling the identification of the most suitable approach for discriminating Parkinson’s disease from healthy controls. Furthermore, our framework incorporated explainable feature selection methods, enhancing both model interpretability and clinical relevance.

Valarmathi et al. [41] proposed a feature-based deep learning framework that combined stacked autoencoders with deep neural networks for Parkinson’s disease detection using voice recordings. Their Feature-Based-Deep Neural Network (FB-DNN) technique achieved excellent performance, reporting an ROC–AUC of 0.956 and an accuracy of 0.9615. Similar to their findings, our study demonstrated strong discriminative performance, achieving comparable or higher ROC–AUC values. However, while Valarmathi et al. focused primarily on a deep learning-based architecture, our work provides a broader evaluation by systematically comparing multiple machine learning models, including KNN, Random Forest, SVM, ANN, and Gradient Boosting. This comprehensive comparison offers deeper insight into model robustness and classifier suitability for Parkinson’s disease detection across different learning paradigms.

Lim et al. [42] examined multimodal biometric features, including voice and facial expressions, and applied several machine learning classifiers. They obtained the ROC-AUC up to 0.90, which is quite lower than the ROC-AUC achieved by our machine learning models, which is approximately 0.99. Using only the voice data, the models achieved a much higher performance compared with their work.

AlNajjar et al. [43] tested different types of feature selection techniques for classification of Parkinson’s disease and concluded that Classification and Regression Trees showed the highest performance. Despite achieving a very high recall, their models failed to report other important metrics. Compared with their models, our models provide high performance across all metrics. AlNajjar et al. had a slightly higher recall than ours.

Costantini et al. reported lower values of accuracy, recall, F1-score, and ROC-AUC than we observed in our study and selected KNN and SVM as models with the highest performance. However, we obtained even higher results for all metrics using these models. Importantly, our study focused on Parkinson’s disease and HS classification, and not multi-condition classification, as was used in the Costantini study [44].

Amran Hossain et al. achieved test accuracy of 0.6228–0.8421, which differs significantly depending on the classifier. For example, KNN reached the highest value of Precision and Recall, but still obtained a poor F1-Score due to the imbalanced distribution of classes. However, in our study, most models achieved relatively high metrics, which means that all of them are equally robust. Moreover, all classifiers showed accuracy above 0.90, namely, ANN 0.9545, KNN 0.9494, Gradient Boosting 0.9442. It should be noted that unlike their models, our ones demonstrate a good balance between precision and recall [45].

Beyond the detection of Parkinson’s disease, machine learning approaches may have broader applications in the differential assessment of related neurological disorders. Speech impairment is not unique to Parkinson’s disease and may also occur in atypical parkinsonian syndromes, essential tremor, multiple system atrophy, progressive supranuclear palsy, and other neurodegenerative conditions. Consequently, voice-based digital biomarkers combined with explainable machine learning techniques may contribute to identifying disease-specific speech patterns and improving diagnostic accuracy. However, further studies involving multiple disease cohorts, larger sample sizes, and external validation datasets are required to determine the clinical utility of these approaches for differential diagnosis and disease monitoring. Therefore, further research involving multiple disease cohorts, larger sample sizes, and external validation datasets is required to evaluate the clinical utility of these approaches for distinguishing Parkinson’s disease from other neurological disorders.

## 5. Conclusions

This study demonstrates that machine learning techniques were capable of effectively distinguishing between individuals diagnosed with Parkinson’s disease and healthy controls using voice recordings. The evaluation metrics, including accuracy, precision, recall, F1-score, and ROC–AUC, indicate consistently strong performance across all models. Notably, most models achieved values exceeding 0.90 for these metrics, reflecting high classification reliability. Among the traditional machine learning approaches, Artificial Neural Networks (ANN) and K-Nearest Neighbors (KNN) exhibited the best overall performance, achieving the highest accuracy and demonstrating strong stability, as evidenced by their low error rates and consistent performance across the 5-fold cross-validation procedure. Furthermore, the application of SHAP-based explainable artificial intelligence enabled the identification of the most informative acoustic features, improving model interpretability and supporting transparent decision-making. An additional strength of this study is the use of repeated voice recordings collected over a six-month period and analyzed through a cross-sectional framework at the recording level, resulting in a substantially larger dataset than would be obtained from a single recording session. Nevertheless, the effective number of independent participants remained limited (8 PD and 8 healthy controls), and therefore the reported performance estimates should be interpreted within the context of a recording-level analysis.

These findings highlight the potential of voice-based digital biomarkers for Parkinson’s disease classification. However, because the present study employed recording-level evaluation, the results should be considered preliminary. Future studies involving larger cohorts and participant-level validation strategies are required before clinical applicability can be established. Future research should focus on validating the proposed framework in larger and more diverse populations, including participants from multiple clinical centers and different linguistic backgrounds. In addition, integrating voice biomarkers with other digital biomarkers, such as gait, handwriting, wearable sensor data, facial expressions, EEG, or neuroimaging measures, may further improve diagnostic performance and clinical utility. Longitudinal studies incorporating disease severity measures, including UPDRS scores, treatment status, and disease progression data, are also needed to evaluate the potential of machine learning models for monitoring disease progression and treatment response over time. Ultimately, the combination of explainable artificial intelligence and digital biomarkers may contribute to the development of clinical decision-support systems for early diagnosis, remote monitoring, and personalized management of Parkinson’s disease.

## 6. Limitations

Several limitations of this study should be acknowledged. First, limited data availability remains a major challenge in digital health research. In the present study, only eight patients with Parkinson’s disease and eight healthy controls were recruited. Nevertheless, the cross-sectional analysis at the recording-level design enabled the collection of 93 voice recordings from Parkinson’s disease patients and 96 voice recordings from healthy controls over a six-month period. Although the raw voice recordings were segmented into 777 speech samples to increase the number of analyzable observations, machine learning approaches generally require substantially larger datasets to ensure robust model development and reliable generalization. Another limitation relates to the classification of healthy controls. The health status of the healthy participants was based on self-report and was not independently verified by a neurologist. Consequently, it cannot be completely excluded that undiagnosed neurological or speech-related conditions were present within the healthy control group. In addition, detailed clinical information was not available for all participants. Although disease severity was partially characterized using the Hoehn and Yahr stage, UPDRS score, and disease duration, other clinically relevant variables, including Parkinson’s disease subtypes, medication status (ON/OFF state), Levodopa Equivalent Daily Dose (LEDD), specific UPDRS subcomponents, and comorbidities, were not consistently available. Therefore, the potential influence of these factors on speech characteristics and machine learning performance could not be assessed. Furthermore, although the mean Hoehn and Yahr stage (1.75 ± 0.59) suggested that most participants were in the early stages of Parkinson’s disease, a more comprehensive clinical assessment by a Parkinson’s disease specialist would have strengthened the interpretation of the findings. Future studies should incorporate more detailed clinical characterization to enable stratified analyses and improve the clinical applicability of voice-based Parkinson’s disease detection models. Another limitation is that the Parkinson’s disease and healthy control groups were not perfectly age-matched, with the Parkinson’s disease group being older on average than the healthy control group. Although age was not included as an input feature in the machine learning models, age-related changes in vocal characteristics may have influenced the observed classification performance. Therefore, the potential confounding effect of age cannot be completely excluded. Future studies should include larger and more closely age-matched cohorts to further evaluate the independent contribution of vocal biomarkers to Parkinson’s disease detection. The important point is that although age and sex were not included in the acoustic-feature models, the sensitivity analysis showed that demographic variables alone had substantial discriminatory power. Therefore, the findings should be validated in a larger age- and sex-matched cohort.

The Decision Tree model exhibited a substantially higher log-loss value than the other evaluated classifiers, indicating poorer probability calibration despite its acceptable classification accuracy. This behavior is common for single Decision Tree models, which may generate overconfident probability estimates due to the hard partitioning of the feature space. Consequently, the probabilistic outputs of the Decision Tree model should be interpreted with caution. Future studies may benefit from the application of probability calibration techniques, such as Platt scaling or isotonic regression, to improve the reliability of predicted probabilities. Finally, although the study was conducted over a six-month period, the present analysis was performed at the recording level rather than the subject level. Consequently, the findings should be interpreted as preliminary and require validation in larger cohorts and independent datasets. Future research should also investigate subject-level validation strategies and longitudinal analyses to further assess the generalizability and clinical utility of voice-based machine learning approaches for Parkinson’s disease detection and monitoring.

## Figures and Tables

**Table 1:** Summary of expected and collected voice recordings during the six-month follow-up period for Parkinson’s disease patients and healthy controls.

Recording Summary	Parkinson’s Disease (*n* = 8)	Healthy Controls (*n* = 8)
Expected recordings	96	96
Actual recordings collected	93	96

**Table 2:** Segment Distribution Per Participant.

Numbers	Participant ID	Group	Segments
1	HC_01	HC	54
2	HC_02	HC	46
3	HC_03	HC	59
4	HC_04	HC	30
5	HC_05	HC	50
6	HC_06	HC	60
7	HC_07	HC	45
8	HC_08	HC	60
9	PD_01	PD	56
10	PD_02	PD	67
11	PD_03	PD	47
12	PD_04	PD	39
13	PD_05	PD	41
14	PD_06	PD	46
15	PD_07	PD	32
16	PD_08	PD	45

**Table 3:** Class-level train–test balance maintained across the five random splits.

Split Number	Train HS	Train PD	Total Train	Test HS	Test PD	Total Test
Split 1–5	283	261	544	121	112	233

**Table 4:** Feature extraction parameters used in the acoustic analysis.

Features	Description	Parameters
F0	Vocal fold vibration rate	pitch_floor = 75.0 pitch_ceiling = 500.0 time_step = 0.01
Shimmer	Measures amplitude fluctuations in vocal cycles	max_amplitude_factor = 1.6
MFCC	Represents spectral envelope of the signal, useful for voice quality analysis	n_mfcc = 1–12 n_fft = 2048 hop_length = 512, n_mels = 40
Linear Prediction Coding (LPC)	Speech samples can be predicted from a small number of previous speech samples	n-LPC = 1–10 frame_length = 0.025 hop_length = 0.010
RPDE	Measures regularity of voice signal	embedding_dim = 2 time_delay = 1 epsilon = 0.05 T_max = 100

**Table 5:** Top 20 acoustic features selected by SHAP-based feature selection.

Rank	Feature Name
1	mfcc_11_mean
2	mfcc_10_mean
3	meanF0
4	mfcc_2_mean
5	mfcc_6_mean
6	stdevF0
7	pitch_mean
8	apq11Shimmer
9	mfcc_12_mean
10	mfcc_delta2_11_std
11	mfcc_delta2_11_mean
12	LPC_2
13	mfcc_0_mean
14	ste_mean
15	mfcc_3_std
16	RPDE
17	mfcc_3_mean
18	mfcc_delta2_1_mean
19	mfcc_delta2_1_std
20	LPC_6

**Table 6:** Hyperparameter tuning of machine learning models.

MODEL	HYPERPARAMETERS
RANDOM FOREST	n_estimators = 400; max_depth = None; min_samples_split = 5; min_samples_leaf = 1; max_features = log2; class_weight = balanced
GAUSSIAN NAÏVE BAYES	var_smoothing = 2.97 × 10^−8^
K-NEAREST NEIGHBORS	n_neighbors = 9; weights = distance; p = 1 (Manhattan distance)
DECISION TREE	criterion = gini; max_depth = 30; min_samples_split = 10; min_samples_leaf = 4
LOGISTIC REGRESSION	penalty = L1; C = 10; solver = liblinear
ARTIFICIAL NEURAL NETWORK (ANN)	hidden_layer_sizes = (64, 64); activation = ReLU; learning_rate_init = 0.01; solver = Adam; bactch_size = 16 max_iter = 150
GRADIENT BOOSTING	n_estimators = 300; learning_rate = 0.2; max_depth = 4
SUPPORT VECTOR MACHINE	kernel = linear; C = 10; probability = True

**Table 7:** System implementation environment.

Resource	Details
CPU	CPU|Intel^®^ Core™ i5-1135G7 @ 2.40 GHz (Intel Corporation, Santa Clara, CA, USA)
RAM	12.67 GB
GPU	4 GB Tesla T4, 15360 MiB
Software	Python 3.10.12 and 3.12.8

**Table 8:** Characteristics of the study participants.

	Healthy Controls (*n* = 8)	Parkinson’s Disease (*n* = 8)
Sex (male/female)	6/2	4/4
Age at enrollment (years)	57.5 ± 7.38	72.37 ± 6.44
Hoehn & Yahr stage of PD	–	1.75 ± 0.59
UPDRS	–	14 ± 4.83
Disease duration (years)	–	7.88 ± 4.49
Voice change (8 patients)	–	8/8

**Table 9:** Age- and sex-only sensitivity analysis for assessing demographic confounding.

Model	Predictors	Validation	AUC	Accuracy	Balanced Accuracy
Logistic Regression	Age + sex	Leave-one-participant-out	0.844	0.75	0.75

**Table 10:** Average test set performance of machine learning models from five-fold cross-validation.

Model	Accuracy	Recall	Precision	F1-Score	ROC-AUC	LOG-LOSS
RANDOM FOREST	0.9313	0.9339	0.9245	0.9289	0.9801	0.2515
NAIVE BAYES	0.8189	0.7714	0.8393	0.8034	0.9004	0.7092
KNN	0.9494	0.9393	0.9548	0.9468	0.9893	0.2510
DECISION TREE	0.8489	0.8321	0.8499	0.8399	0.9021	2.8687
LOGISTIC REGRESSION	0.8704	0.8554	0.8744	0.8638	0.9469	0.3077
ANN	0.9545	0.9500	0.9551	0.9525	0.9882	0.2552
GRADIENT BOOSTING	0.9442	0.9357	0.9481	0.9415	0.9841	0.3816
SVM	0.8652	0.8446	0.8724	0.8577	0.9403	0.3122

## Data Availability

The datasets generated and analyzed during the current study are available from the corresponding author upon reasonable request and subject to approval by the relevant ethical and institutional regulations. Due to privacy and confidentiality considerations associated with clinical voice recordings from human participants, the raw voice data cannot be made publicly available without appropriate authorization. To promote transparency and reproducibility, the source code used for machine learning model development, and performance evaluation has been made publicly available through GitHub: https://github.com/mehdi1366-com (I checked this online resource on 10 August 2026.).
